# Maternal occupational exposure and oral clefts in offspring

**DOI:** 10.1186/s12940-017-0294-5

**Published:** 2017-08-04

**Authors:** Nynke Spinder, Jorieke E. H. Bergman, H. Marike Boezen, Roel C. H. Vermeulen, Hans Kromhout, Hermien E. K. de Walle

**Affiliations:** 1 Department of Epidemiology, University of Groningen, University Medical Center Groningen, HPC FA 40, P.O. Box 30.001, 9700 RB Groningen, the Netherlands; 2 Department of Genetics, University of Groningen, University Medical Center Groningen, HPC CB 51, P.O. Box 30.001, 9700 RB Groningen, the Netherlands; 3 Division of Environmental Epidemiology, Institute for Risk Assessment Science, Utrecht University, Postbox 80178, 3508 TD Utrecht, the Netherlands

**Keywords:** Biological dust, Congenital anomalies, Job-exposure matrix, Metals, Mineral dust, Occupational exposure, Pesticides, Solvents, Teratology

## Abstract

**Background:**

Previous studies suggest that periconceptional maternal occupational exposure to solvents and pesticides increase the risk of oral clefts in the offspring. Less is known about the effect of occupational exposure to metals, dust, and gases and fumes on development of oral clefts.

**Methods:**

This case-malformed control study used data from a population-based birth defects registry (Eurocat) of children and foetuses born in the Northern Netherlands between 1997 and 2013. Cases were defined as non-syndromic oral clefts. The first control group had chromosomal/monogenic defects, and the second control group was defined as non-chromosomal/non-monogenic malformed controls. Maternal occupational exposure was estimated through linkage of mothers’ occupation with a community-based Job Exposure Matrix (JEM). Multivariate logistic regression was used to estimate the effect of occupational exposures. Odds ratios were adjusted (aORs) for relevant confounders.

**Results:**

A total of 387 cases, 1135 chromosomal and 4352 non-chromosomal malformed controls were included in this study. Prevalence of maternal occupational exposures to all agents was 43.9% and 41.0%/37.7% among cases and controls, respectively. Oral clefts had significantly increased ORs of maternal occupational exposure to pesticides (aOR = 1.7, 95% confidence interval [CI] 1.0–3.1) and dust (aOR = 1.3, 95% CI 1.1–1.6) when using non-chromosomal controls. Subgroup analysis for CL(P) stratified by gender showed a significantly increased risk for male infants exposed to ‘other solvents’ and exposure to mineral dust for female infants.

**Conclusion:**

Our study showed that maternal occupational exposure to pesticides and dust are risk factors for oral clefts in the offspring. Larger studies are needed to confirm this finding.

## Background

Oral clefts are one of the most common congenital anomalies in the Netherlands with a prevalence of 2.1 per 1000 live births [1]*.* Oral clefts are complex malformations that result from failure of fusion of the lip or palate. Because of different developmental origins, oral clefts can be classified as cleft palate (CP) or cleft lip with or without palate (CL(P)). Oral clefts have a large impact on the affected individuals, their parents and on the community in terms of physical and emotional well-being, and medical costs [2]. The etiology of oral clefts is not fully understood, but involves genetic as well as environmental factors. Several environmental factors during pregnancy have been associated with an increased risk of oral clefts in the offspring, including maternal smoking [3], maternal alcohol consumption [4] and high maternal pre-pregnancy body mass index (BMI) (>30 kg/m^2^) [5–7]. There is no consensus on whether folic acid is protective or might be a risk factor for oral clefts [8].

Participation of Dutch women in the labour market has increased substantially over the last two decades [9]. Therefore, it is important to examine exposure to various teratogenic factors in the workplace. Large population based case-control studies suggest a relationship between exposure to organic solvents and oral clefts [10–17], whereas one other study did not find a higher risk of oral clefts in the offspring after maternal occupational exposure to solvents [18].

Several studies have investigated maternal occupational exposure to pesticides and risk of oral clefts in the offspring. Romitti et al. performed a meta-analysis and concluded that maternal exposure to pesticides in general is associated with a small increased risk of oral clefts in the offspring [19]. More recently, Yang et al. assessed residential exposure to specific agricultural pesticides in an area with high rates of pesticide use and concluded that there was a positive relationship between herbicide exposure and oral clefts, especially among female infants [20].

There is one previous study that suggested an association between maternal occupational exposure to metals and oral clefts in the offspring [21]. As far as we know, there is no literature concerning occupational exposure to mineral and organic dust, and gases and fumes in relation to the occurrence of oral clefts. However, since these exposures often occur in the same workplace as exposure to solvents and pesticides, these exposures were also taken into account in this study.

The objective of this case-malformed control study was to examine the association between maternal occupational exposure to, in particular solvents and pesticides, but also to metals, mineral and organic dusts, and gases and fumes during the periconceptional period and risk of oral clefts in the offspring.

## Methods

### Study design and population

To examine the possible association between maternal occupational exposure and oral clefts in the offspring a case-malformed control study was performed. Cases and malformed controls were selected from the European Registration of Congenital Anomalies and Twins database of the Northern Netherlands (Eurocat NNL). This population-based registry has been monitoring congenital anomalies in about 18,000 births annually in the provinces of Groningen, Friesland and Drenthe since 1981. In addition to live births (up to 10 years of age at notification), stillbirths, miscarriages and terminated pregnancies because of a congenital anomaly, are registered in the database. Children and foetuses are only registered in Eurocat NNL after parents give informed consent. In general, the informed consent rate is around 80% for all types of congenital anomalies.

Coding and classification of congenital anomalies are performed according to Eurocat guidelines [22]. In this study, Eurocat NNL data of children and foetuses born from 1997 until 2013 was used.

### Data collection

Since 1997, parents have been asked to complete a written questionnaire to supply information about the pregnancy. The questionnaire includes a question about maternal occupation and the workplace (e.g. the company where the mother worked) at the beginning of the pregnancy. In addition, information is gathered concerning medical history, demographic characteristics and maternal pre-pregnancy weight and height. For smoking habits, alcohol consumption, and the use of medication, information is gathered from three months before pregnancy until the end of pregnancy. After parental consent, data on prescribed medication is retrieved from the pharmacy. Ambiguities in the questionnaire, actual use of medication and for which period it was used, were verified in a telephone interview with the mother.

### Definition of cases and controls

Cases were defined as non-syndromic clefts, either occurring isolated or together with other major congenital anomalies. Children with a Pierre Robin sequence were included in the case group. International Classification of Diseases 9th revision (ICD-9, 749) was used for births up until 2001 and the ICD-10 classification (Q35-Q37) was used for births since 2002. A total of 679 cases with an oral cleft were selected for this study. Cases with a cleft that were also labelled as having a chromosomal or monogenic disorder were excluded (*n* = 89), because these clefts may be part of that specific syndrome. Additionally, cases with anencephaly, arhinencephaly and holoprosencephaly were excluded (*n* = 9) because these anomalies are often associated with oral clefts. In total, 95 cases (14%) were excluded because mothers’ occupation was unknown (e.g. the questionnaire was not returned). In this study only mothers with a paid job were included, which led to an exclusion of 99 cases (e.g. housewives).

Non-malformed children are not registered in the Eurocat database. Infants and foetuses born with chromosomal/monogenic disorders, not accompanied by oral clefts, were used as controls, because the etiology of these malformations is known. In total, 1764 chromosomal controls were selected for this study. We excluded 357 controls (20%) because mothers’ occupation was unknown and another 272 controls were excluded because their mothers had no paid job. Hereafter we refer to this group as chromosomal controls.

Analyses were performed with a second control group, because chromosomal controls can introduce bias through higher maternal age. This second control group is defined as all other babies/foetuses registered in Eurocat with non-chromosomal/non-monogenic disorders, and no malformation accompanied by an oral cleft. A total of 6847 babies/foetuses were selected for the non-chromosomal malformed control group. Because mothers’ occupation was unknown, 1626 controls (24%) were excluded. Furthermore, 869 controls were excluded because mother had no paid job. Hereafter we refer to this group as non-chromosomal controls.

This resulted in a total of 387 cases, 1135 chromosomal controls and 4352 non-chromosomal controls. Cases were further subdivided in a group of CP (*n* = 124) and a group of CL(P) (*n* = 263).

### Exposure assessment

A community-based JEM (ALOHA+ JEM) is applied to translate self-reported information about mothers’ occupation during the periconceptional period (three months before conception through the first trimester) into occupational exposures to solvents, pesticides, metals and more generic categories like mineral and organic dust, and gases and fumes. The ALOHA+ JEM is built specifically for use in community-based studies [23]. Given that specific occupational exposures are relatively rare in the general population, specificity in exposure assignment was preferred over sensitivity when elaborating the ALOHA+ JEM [24].

Jobs were coded by two of the authors (NS and HK) into the International Standard Classification of Occupations 1988 (ISCO88) without knowledge of case/control status [25]. The ALOHA+ JEM assigned occupational exposure to solvents (aromatic, chlorinated and other [e.g. alkanes, alcohols, and esters]), pesticides (fungicides, herbicides and insecticides), metals, dust (organic and mineral), and gases and fumes. Based on the mothers’ occupation, the JEM assigned no (0), low (1) or high (2) exposure to solvents, pesticides, metals, dust, and gases and fumes. For mothers who had two or more jobs with different exposures, the highest exposure category was selected.

### Variable definition

Potential confounders applied in our analyses were child sex (boy or girl), number of babies/foetuses delivered (1 or ≥2), previous births (0, 1 or ≥2 births), maternal age at delivery (15–19, 20–24, 25–29, 30–39, ≥40 years old), maternal BMI (underweight [<18.5 kg/m^2^], normal [18.5–25 kg/m^2^], overweight [25–30 kg/m^2^], obese [>30 kg/m^2^]), maternal education level (low [primary school, lower vocational education, pre-vocational education], middle [secondary vocational education, general secondary education or pre-university education] or high [higher professional education or academic education]), maternal smoking (no, yes/some period during pregnancy), maternal alcohol use during pregnancy (no, yes/some period during pregnancy), folic acid use (no/wrong period, yes/periconceptional period [400 μg folic acid/day from 4 weeks before until 8 weeks after conception [26]), fertility problems (no, yes [self-reported fertility problems or fertility treatment]) and positive family history (yes/no). A positive family history means a first-degree family member with the same condition as the baby/foetus under study, e.g. if a child has an oral cleft, the family history is positive when a first degree family member has an oral cleft as well.

### Statistical analyses

The associations between specific maternal occupational exposures and oral clefts were assessed using univariate and multivariate logistic regression models to estimate crude odds ratios (OR) and adjusted ORs. We adjusted multivariate models for potential confounders, based on significance using Chi Square tests. Confounders for the analyses with chromosomal controls were child sex, maternal age at delivery, pre-pregnancy BMI, education level, smoking and alcohol use during pregnancy, and family history. Analyses with non-chromosomal controls were corrected for child sex and previous births as confounders. Separate subgroup analyses were conducted for CP and CL(P) alone compared with both control groups.

From literature is known that the prevalence of CL(P) is higher among male infants. Therefore, an additional analysis was performed stratified by child’s gender. Due to the small number of mothers with high exposure, low and high exposure were merged into one ‘any exposure’ group for all types of occupational exposures. Additionally, for specific exposure categories with a high prevalence of exposed cases, it was possible to evaluate no, low, and high exposure categories separately. *P*-values of <0.05 were considered statistically significant. Statistical Package for the Social Sciences version 22 (SPSS V22) was used to perform all analyses.

## Results

The baseline characteristics of 387 cases, 1135 chromosomal controls and 4352 non-chromosomal controls are presented in Table 1. Among cases there was a significant excess of males compared to chromosomal controls. Case mothers had a younger age at delivery, a higher BMI and their education level was lower. Furthermore, they smoked more often, used alcohol less often, and had less often a positive family history. The significant differences in baseline characteristics between oral clefts and chromosomal controls apply as well when CL(P) and chromosomal controls were compared, except for pre-pregnancy BMI. There were no significant differences in baseline characteristics between CP and chromosomal controls.

**Table 1:** Baseline characteristics of cases (all oral clefts, cleft palate (CP), cleft lip with/without cleft palate CL(P)) compared with two malformed control groups

	Chromosomal controls^a^		Non-chromosomal controls^b^		All oral cleft				CP				CL(P)			
	n	%	n	%	n	%	*p*-value^c^	*p*-value^d^	n	%	*p*-value^c^	*p*-value^d^	n	%	*p*-value^c^	*p*-value^d^
	1135		4356		387				124				263			
Child sex							**<0.001**	0.21			0.83	0.43			**<0.001**	**0.02**
Boy	547	48.2	2373	54.5	227	58.7			61	49.2			166	63.1		
Girl	588	51.8	1970	45.3	160	41.3			63	50.8			97	36.9		
Unknown	0		9		0				0				0			
Number of babies/foetuses delivered							0.31	0.98			0.09	0.30			0.86	0.50
1	1089	96.0	4104	94.8	366	94.8			115	92.7			251	95.8		
> 1	45	4.0	223	5.2	20	5.2			9	7.3			11	4.2		
Unknown	1		25		1				0				1			
Previous births							0.24	**0.01**			0.60	0.11			0.36	0.05
0	476	42.3	2313	53.2	180	46.6			57	46.0			123	46.9		
1	461	40.9	461	34.6	141	36.5			45	36.3			96	36.6		
≥ 2	189	16.8	189	12.1	65	16.8			22	17.7			43	16.4		
Unknown	9		8		1				0				1			
Maternal age at delivery							**<0.001**	0.66			0.09	0.40			**<0.001**	0.66
15–19	4	0.4	14	0.3	2	0.5			0				2	0.8		
20–24	55	4.8	369	8.5	24	6.2			8	6.5			16	6.1		
25–29	299	26.3	1458	33.5	132	34.3			43	35.0			89	34.0		
30–34	407	35.9	1770	40.7	154	40.0			47	38.2			107	40.8		
35–39	284	25.0	651	15.0	63	16.4			19	15.4			44	16.8		
> 40	86	7.6	86	2.0	10	2.6			6	4.9			4	1.5		
Unknown	0		0		2				1				1			
Pre-pregnancy BMI (kg/m^2^)							**0.03**	0.21			0.19	0.49			0.09	0.29
< 18.5	30	2.8	112	2.7	12	3.2			2	1.7			10	3.9		
18.5–25	721	66.3	2713	64.6	224	59.1			71	59.2			153	59.1		
25–30	258	23.7	975	23.2	101	26.6			33	27.5			68	26.3		
> 30	78	7.2	402	9.6	42	11.1			14	11.7			28	10.8		
Unknown	48		150		8				4				4			
Education level							**0.01**	0.84			0.59	0.72			**0.01**	0.56
Low	151	13.8	545	12.8	51	13.5			15	12.4			36	14.0		
Middle	462	42.2	2137	50.3	193	51.1			57	47.1			136	52.9		
High	481	44.0	1568	36.9	134	35.4			49	40.5			85	33.1		
Unknown	41		102		9				3				6			
Smoking during pregnancy							**0.02**	0.47			0.72	0.49			**0.01**	0.18
No	89	81.0	3321	77.0	291	75.4			98	79.7			193	73.4		
Yes	210	19.0	992	23.0	95	24.6			25	20.3			70	26.6		
Unknown	28		39		1				1				0			
Alcohol during pregnancy							**0.02**	0.16			0.25	0.62			**0.03**	0.17
No	811	73.3	3276	76.1	306	79.3			96	78.0			210	79.8		
Yes	296	26.7	1029	23.9	80	20.7			27	22.0			53	20.2		
Unknown	28		47		1				1				0			
Folic acid use							0.64	0.84			0.67	0.52			0.34	0.49
No	224	21.3	844	20.5	77	20.1			28	23.0			49	18.8		
Yes	830	78.7	3265	79.5	306	79.9			94	77.0			212	81.2		
Unknown	81		243		4				2				2			
Fertility problems							0.39	0.29			0.73	0.11			0.19	0.84
No	889	81.0	3634	85.0	316	82.9			98	79.7			218	84.5		
Yes	209	19.0	643	15.0	65	17.1			25	20.3			40	15.5		
Unknown	37		75		6				1				5			
Positive family history^e^							**0.003**	0.08			0.17	0.60			**0.005**	0.07
No	960	85.0	3819	88.0	352	91.0			111	89.5			241	91.6		
Yes	170	15.0	523	12.0	35	9.0			13	10.5			22	8.4		
Unknown	4		10		0				0				0			

^a^chromosomal/monogenic controls (not accompanied with oral clefts), ^b^non-chromosomal/non-monogenic malformed controls (not accompanied with oral clefts), ^c^ *p*-value comparing cases with chromosomal controls, ^d^ *p*-value comparing cases with non-chromosomal controls, ^e^positive family history when first degree family member has same condition as child under study. Bold values represent significant values (*p* < 0.05)

None of these significant differences were observed when cases were compared with non-chromosomal controls, except the excess of males in the CL(P) group. Mothers with a child with an oral cleft had significantly more previous births.

The prevalence of estimated occupational exposure to any of the agents considered was 43.9% among case mothers, 41.0% among mothers of chromosomal controls (Table 2), and 37.7% among non-chromosomal controls (Table 3). Prevalence of maternal exposure to solvents was similar among cases and controls. The most frequent type of solvent exposure was exposure to ‘other solvents’. Mothers exposed to ‘other solvents’ were mainly working in healthcare. The prevalence of occupational exposure to pesticides was low, but was higher among cases than controls (3.6% versus 2.4% for chromosomal controls and 2.0% for non-chromosomal controls). Maternal occupational exposure to organic dust occurred most frequent, with case mothers being more often exposed to organic dust than chromosomal/non-chromosomal controls (36.7% versus 32.6%/29.6%). Mothers exposed to organic dust were working in e.g. healthcare or agriculture.

**Table 2:** Prevalence exposures and association between periconceptional maternal occupational exposure and all oral clefts, cleft palate (CP), and cleft lip with/without cleft palate (CL(P)) using chromosomal/monogenic controls

Exposure	Diagnosis	Prevalence exposure					
		n	%	OR	95% CI	aOR^a^	95%CI
Any agent	Chromosomal control	465	41.0	Ref		Ref	
	All oral cleft	170	43.9	1.1	0.9–1.4	1.0	0.8–1.3
	CP	42	33.9	0.7	0.5–1.1	0.7	0.5–1.1
	CL(P)	128	48.7	1.4	1.0–1.8	1.2	0.9–1.6
Solvents	Chromosomal control	281	24.8	Ref			
	All oral cleft	103	26.6	1.1	0.8–1.4	1.0	0.8–1.4
	CP	29	23.4	0.9	0.6–1.4	0.9	0.6–1.4
	CL(P)	74	28.1	1.2	0.9–1.6	1.1	0.8–1.5
Aromatic solvents	Chromosomal control	50	4.4	Ref		Ref	
	All oral cleft	17	4.4	1.0	0.6–1.8	1.1	0.6–1.8
	CP	5	4.0	0.9	0.4–2.3	1.0	0.4–2.6
	CL(P)	12	4.6	1.0	0.5–2.0	1.1	0.5–2.0
Chlorinated solvents	Chromosomal control	53	4.7	Ref		Ref	
	All oral cleft	18	4.7	1.0	0.6–1.7	1.0	0.5–1.7
	CP	4	3.2	0.7	0.2–1.9	0.7	0.2–1.9
	CL(P)	14	5.3	1.1	0.6–2.1	1.1	0.6–2.0
Other solvents	Chromosomal control	263	23.2	Ref		Ref	
	All oral cleft	99	25.6	1.2	0.9–1.5	1.1	0.8–1.4
	CP	28	22.6	1.0	0.6–1.5	0.9	0.6–1.5
	CL(P)	71	27.0	1.2	0.9–1.7	1.2	0.8–1.6
Pesticides	Chromosomal control	27	2.4	Ref		Ref	
	All oral cleft	14	3.6	1.5	0.8–3.0	1.5	0.8–3.0
	CP	5	4.0	1.7	0.7–4.6	1.7	0.6–4.6
	CL(P)	9	3.4	1.5	0.7–3.1	1.4	0.6–3.1
Fungicides	Chromosomal control	23	2.0	Ref		Ref	
	All oral cleft	13	3.4	1.7	0.8–3.4	1.7	0.8–3.5
	CP	5	4.0	2.0	0.8–5.4	2.1	0.7–5.7
	CL(P)	8	3.0	1.5	0.7–3.4	1.5	0.6–3.4
Herbicides	Chromosomal control	15	1.3	Ref		Ref	
	All oral cleft	6	1.6	1.2	0.5–3.1	1.2	0.4–3.1
	CP	1	0.8	0.6	0.1–4 6	0.6	0.1–4.6
	CL(P)	5	1.9	1.4	0.5–4.0	1.3	0.5–3.9
Insecticides	Chromosomal control	25	2.2	Ref		Ref	
	All oral cleft	14	3.6	1.7	0.9–3.2	1.7	0.8–3.3
	CP	5	4.0	1.9	0.7–5.0	1.8	0.7–5.0
	CL(P)	9	3.4	1.6	0.7–3.4	1.5	0.7–3.3
Heavy metals	Chromosomal control	14	1.3	Ref		Ref	
	All oral cleft	4	1.0	0.8	0.3–2.6	0.6	0.2–2.3
	CP	1	0.8	0.7	0.1–5.0	0.6	0.1–5.0
	CL(P)	3	1.1	0.9	0.3–3.2	0.8	0.2–3.1
Dust	Chromosomal control	385	33.9	Ref		Ref	
	All oral cleft	146	37.7	1.2	0.9–1.5	1.1	0.9–1.4
	CP	37	28.8	0.8	0.5–1.2	0.8	0.5–1.2
	CL(P)	109	41.4	1.3	0.9–1.7	1.3	0.9–1.7
Organic dust	Chromosomal control	370	32.6	Ref		Ref	
	All oral cleft	142	36.7	1.2	0.9–1.5	1.2	0.9–1.4
	CP	36	29.0	0.8	0.6–1.3	0.8	0.5–1.2
	CL(P)	106	40.3	1.4	1.1–1.8	1.3	0.9–1.7
Mineral dust	Chromosomal control	111	9.8	Ref		Ref	
	All oral cleft	40	10.3	1.1	0.7–1.6	1.1	0.7–1.6
	CP	9	7.3	0.7	0.4–1.5	0.8	0.4–1.6
	CL(P)	31	11.8	1.2	0.8–1.9	1.2	0.8–2.0
Gases and fumes	Chromosomal control	353	31.1	Ref		Ref	
	All oral cleft	126	32.6	1.1	0.8–1.4	1.0	0.8–1.3
	CP	29	23.4	0.7	0.4–1.0	0.6	0.4–1.0
	CL(P)	97	36.9	1.3	1.0–1.7	1.2	0.9–1.6

^a^Odds ratio adjusted for child sex, maternal age at delivery, pre-pregnancy body mass index, education level, smoking and alcohol use during pregnancy, and family history

**Table 3:** Prevalence exposures and association between periconceptional maternal occupational exposure and all oral clefts, cleft palate (CP), and cleft lip with/without cleft palate (CL(P)) using non-chromosomal/non-monogenic malformed controls

Exposure	Diagnosis	Prevalence exposure					
		n	%	OR	95% CI	aOR^a^	95%CI
Any agent	Non-chromosomal control	1642	37.7	Ref		Ref	
	All oral cleft	170	43.9	**1.3**	**1.0–1.6**	**1.3**	**1.0–1.6**
	CP	42	33.9	0.8	0.6–1.2	0.8	0.6–1.2
	CL(P)	128	48.7	**1.6**	**1.2–2.0**	**1.5**	**1.2–2.0**
Solvents	Non-chromosomal control	1075	24.7	Ref			
	All oral cleft	103	26.6	1.1	0.9–1.4	1.1	0.9–1.4
	CP	29	23.4	0.9	0.6–1.4	0.9	0.6–1.4
	CL(P)	74	28.1	1.2	0.9–1.6	1.2	0.9–1.6
Aromatic solvents	Non-chromosomal control	140	3.2	Ref		Ref	
	All oral cleft	17	4.4	1.4	0.8–2.3	1.4	0.8–2.3
	CP	5	4.0	1.3	0.5–3.1	1.3	0.5–3.1
	CL(P)	12	4.6	1.4	0.8–2.6	1.5	0.8–2.7
Chlorinated solvents	Non-chromosomal control	190	4.4	Ref		Ref	
	All oral cleft	18	4.7	1.1	0.7–1.8	1.1	0.7–1.8
	CP	4	3.2	0.7	0.3–2.0	0.7	0.3–2.0
	CL(P)	14	5.3	1.2	0.7–2.2	1.3	0.7–2.2
Other solvents	Non-chromosomal control	1042	23.9	Ref		Ref	
	All oral cleft	99	25.6	1.1	0.9–1.4	1.1	0.9–1.4
	CP	28	22.6	0.9	0.6–1.4	0.9	0.6–1.4
	CL(P)	71	27.0	1.2	0.9–1.6	1.2	0.9–1.6
Pesticides	Non-chromosomal control	88	2.0	Ref		Ref	
	All oral cleft	14	3.6	**1.8**	**1.0–3.2**	**1.7**	**1.0–3.1**
	CP	5	4.0	2.0	0.8–5.1	1.9	0.8–4.8
	CL(P)	9	3.4	1.7	0.9–3.4	1.7	0.8–3.4
Fungicides	Non-chromosomal control	70	1.6	Ref		Ref	
	All oral cleft	13	3.4	**2.1**	**1.2–3.9**	**2.0**	**1.1–3.7**
	CP	5	4.0	**2.6**	**1.0–6.5**	2.4	0.9–6.0
	CL(P)	8	3.0	1.8	0.9–3.6	1.9	0.9–4.0
Herbicides	Non-chromosomal control	36	0.8	Ref		Ref	
	All oral cleft	6	1.6	1.9	0.8–4.5	1.8	0.8–4.4
	CP	1	0.8	1.0	0.1–7 2	0.9	0.1–7.0
	CL(P)	5	1.9	2.3	0.9–6.0	2.3	0.9–5.9
Insecticides	Non-chromosomal control	84	1.9	Ref		Ref	
	All oral cleft	14	3.6	**1.9**	**1.1–3.4**	**1.8**	**1.0–3.2**
	CP	5	4.0	2.1	0.9–5.4	2.0	0.8–5.0
	CL(P)	9	3.4	1.8	0.9–3.6	1.7	0.9–3.5
Heavy metals	Non-chromosomal control	44	1.0	Ref		Ref	
	All oral cleft	4	1.0	1.0	0.4–2.9	1.1	0.4–3.0
	CP	1	0.8	0.8	0.1–5.8	0.9	0.1–6.3
	CL(P)	3	1.1	1.1	0.3–3.7	1.2	0.4–3.8
Dust	Control	1346	30.9	Ref		Ref	
	All oral cleft	146	37.7	**1.4**	**1.1–1.7**	**1.3**	**1.1–1.6**
	CP	37	28.8	1.0	0.6–1.4	0.9	0.6–1.4
	CL(P)	109	41.4	**1.6**	**1.2–2.0**	**1.5**	**1.2–2.0**
Organic dust	Non-chromosomal control	1288	29.6	Ref		Ref	
	All oral cleft	142	36.7	**1.4**	**1.1–1.7**	**1.3**	**1.1–1.7**
	CP	36	29.0	1.0	0.7–1.4	1.0	0.6–1.4
	CL(P)	106	40.3	**1.6**	**1.2–2.1**	**1.6**	**1.2–2.0**
Mineral dust	Non-chromosomal control	396	9.1	Ref		Ref	
	All oral cleft	40	10.3	1.2	0.8–1.6	1.1	0.8–1.6
	CP	9	7.3	0.8	0.4–1.6	0.8	0.4–1.5
	CL(P)	31	11.8	1.3	0.9–2.0	1.3	0.9–1.9
Gases and fumes	Non-chromosomal control	1521	34.9	Ref		Ref	
	All oral cleft	126	32.6	0.8	0.7–1.1	0.9	0.7–1.1
	CP	29	23.4	**0.6**	**0.4–0.9**	**0.6**	**0.4–0.9**
	CL(P)	97	36.9	1.1	0.8–1.4	1.1	0.8–1.4

^a^Odds ratio adjusted for child sex, and previous births. Bold values represent significant values

Table 2 shows the adjusted ORs of maternal occupational exposure. The aORs for maternal occupational exposure to solvents, metals, dust, and gases and fumes did not increase significantly when using chromosomal controls.

When using non-chromosomal controls, aORs increased significantly for maternal occupational exposure to pesticides and dust (Table 3). The highest aORs were found for fungicides and insecticides (aOR = 2.0, 95% CI 1.1–3.7 and aOR = 1.8, 95% CI 1.0–3.2, respectively). The aOR for dust, especially organic dust, increased significantly (aOR = 1.3, 95% CI 1.1–1.7). The significant changes were also observed for organic dust in the CL(P) group.

Additional analyses with CL(P) cases were performed stratified by child sex. The aOR for periconceptional exposure to ‘other solvents’ increased for male infants (aOR = 1.5, 95% CI 1.1–2.1, data not shown in table) using non-chromosomal controls. The aOR for occupational herbicide exposure in relation to CL(P) increased for female infants (aOR = 3.8, 95% CI 1.1–13.4, data not shown in table). However, this was only based on three exposed cases. Mineral dust exposure was associated with CL(P) for females as well (aOR = 2.0, 95% CI 1.2–3.5, data not shown in table).

For exposure categories with high prevalence in this study (‘other solvents’, organic dust, and gases and fumes), additional analyses were performed for all three exposure intensity categories (no, low, and high). The number of high exposed cases was respectively 10, 11, and 4 cases. The aOR for cases with low exposure to ‘other solvents’ was 1.1 (95% CI 0.8–1.5), and increased to 1.5 (95% CI 0.8–3.0) for cases with high exposure (data not shown in table). For occupational exposure to organic dust the same trend is observed. The aOR increased from 1.3 (95% CI 1.1–1.6) for low exposure, to 1.7 (95% CI 0.9–3.2) for high exposure (data not shown in table). No trend of increased is observed OR for occupational exposure to gases and fumes. However, all ORs did not increase significantly.

## Discussion

Results from this population-based case-malformed control study indicate an effect for maternal periconceptional occupational exposure to fungicides, insecticides, and organic dust on the risk of oral clefts in the offspring. Male infants have an increased risk on CL(P) when mothers are occupational exposed to ‘other solvents’. Females have an increased on CL(P) when mothers are exposed to mineral dust. This study shows overall no increased risk of clefts in the offspring when mothers are periconceptionally occupational exposed to solvents, metals, and gases and fumes.

The association between maternal pesticide exposure and oral clefts in the offspring is described previously. A meta-analysis from 2007, that examined the association between occupational exposure to pesticides during pregnancy and oral clefts, showed a significant increased risk of oral clefts (OR = 1.37, 95% CI 1.04–1.81) [19]. This is comparable to our study, where we find slightly higher OR of 1.7, 95% CI 1.0–3.1. Most mothers exposed to pesticides in our study were working in agriculture. A Finnish study examined the association between working in agriculture and oral clefts in the offspring [27]. They found a comparable increased OR of oral clefts in the offspring among mothers working in agriculture during the first trimester of their pregnancy (OR = 1.9, 95% CI 1.1–3.5).

Furthermore, we observed an association between maternal exposure to dust and oral clefts in the offspring. Despite the fact that occupational exposure to dust is common at the workplace, no studies are known about the relation between occupational dust exposure and congenital anomalies in the offspring.

In our study we found no association between maternal occupational exposure to solvents and oral clefts in the main analyses. However, in the additional analyses an association is found between maternal occupational exposure to ‘other solvents’ and CL(P) in male infants only. Our finding is in line with one study from the USA that reported no association [18], but it is in contrast with multiple studies published since 2000 that did report an association between maternal occupational exposure to solvents and oral clefts [10–16]. Most of these studies have been performed in France and the USA and used occupational hygienists, who assessed exposure to specific solvents case-by-case based on detailed standardized interviews in which mothers were asked about job titles and descriptions of the job. The method of classifying occupational exposure by industrial hygienists is more specific and accurate than use of a JEM. However, there is a prospective study, using self-reported exposure assessment as well as a JEM, which reports a significant increased risk of oral clefts in the offspring for mothers exposed to solvents [12]. Inconsistencies could also be due to different definitions of solvent exposure.

We found no significant association between maternal occupational exposure to metals and oral clefts, whereas the study of Hao et al. [21] did find a significant association (OR = 5.67, 95% CI 1.34–24.09). In our study the prevalence of exposure was very low compared to the Chinese study (0.8% in our CP group versus 8.8% in Hao et al.). No other studies have investigated metal exposure in relation to oral clefts.

Finally, we observed no association between maternal occupational exposure to gases and fumes, which we analysed because these are often co-exposures in women exposed to pesticides, solvents and metals.

### Strengths and limitations

A major strength of this study is the use of data from the population-based Eurocat registry. Ascertainment of oral cleft cases by Eurocat NNL was virtually complete for birth years 1997–2009, with a consent rate for registration of over 90% [8].Data in the Eurocat NNL database are of high quality and congenital anomalies are classified according to high standards and ICD codes. This made it possible to accurately distinguish between isolated clefts, clefts occurring together with other major congenital anomalies and syndromic clefts. Moreover, because both cases and both control groups had anomalies, recall bias is not expected to play a role in our study design.

Another strength is the use of the ALOHA+ JEM. The benefit of using a JEM is that it avoids recall bias since the mother is not directly asked about her occupational exposure during pregnancy. Besides, results in occupational exposure estimates are that are less prone to differential misclassification of exposure compared to self-reported exposures [24, 28].

The Eurocat NNL questionnaire includes questions about job title and workplace during pregnancy, but did not include questions about the actual job tasks that were performed. It is therefore possible that women avoided certain activities during the periconceptional period in order to decrease exposure to potential teratogenic agents. Their actual exposure could therefore have been lower or absent from what was assigned by the JEM based on their job. Another limitation of using a JEM, compared to expert assessment, is that JEMs have often low sensitivity. Partly, this low sensitivity is due to the variability in exposure across time which is not taken into account by the JEM [29].

In our study a relatively low numbers of cases are exposed to pesticides. This has resulted in a lower power. Besides, our study could not address exposure intensity for all subcategories of exposure as assigned by the JEM (low or high exposure) separately in our analyses, due to the low numbers of highly exposed women. This precluded an exposure-response evaluation.

Finally, we used malformed controls and could therefore not compare with healthy children. It is known that occupational exposure to pesticides is possibly associated with chromosomal aberrations [30]. Furthermore, residential exposure to solvents or metals has been suggested to be associated with an increased risk of chromosomal anomalies in the offspring of older women [31]. Given our design, if these associations between occupational exposure and chromosomal anomalies would have been present, this would have resulted in attenuated risk estimates of maternal occupational exposures for the risk of oral clefts in the offspring.

## Conclusion

Our study indicates that maternal periconceptional occupational exposure to pesticides and dust are risk factors for oral clefts, in particular exposure to fungicides, insecticides and organic dust is associated with an increased risk for cleft palate in the offspring. Occupational maternal exposure to ‘other solvents’ gives an increased risk of CL(P) in male offspring, whereas mineral dust is associated with CL(P) in female offspring. Exposure to solvents, metals, and gases and fumes are not shown to be associated with oral clefts in the offspring. More data are needed to identify whether the association between periconceptional occupational maternal solvents, pesticides, and dust exposure and cleft palate in the offspring is causal.

## Abbreviations

aOR: Adjusted odds ratio
BMI: Body mass index
CI: Confidence interval
CL(P): Cleft lip with or without palate
CP: Cleft palate
Eurocat NNL: European Registration of Congenital Anomalies and Twins database of the Northern Netherlands
JEM: Job Exposure Matrix
OR: Odds ratio
